# Both Carboplatin and Bevacizumab Improve Pathological Complete Remission Rate in Neoadjuvant Treatment of Triple Negative Breast Cancer: A Meta-Analysis

**DOI:** 10.1371/journal.pone.0108405

**Published:** 2014-09-23

**Authors:** Xiao-song Chen, Ying Yuan, David H. Garfield, Jia-yi Wu, Ou Huang, Kun-wei Shen

**Affiliations:** 1 Comprehensive Breast Health Center, Ruijin Hospital Shanghai Jiaotong University School of Medicine, Shanghai, China; 2 Department of Radiology, Shanghai Ninth People’s Hospital, Affiliated to Shanghai Jiaotong University School of Medicine, Shanghai, China; 3 University of Colorado Comprehensive Cancer Center, Aurora, Colorado, United States of America; Yale University, United States of America

## Abstract

Triple negative breast cancer (TNBC) is associated with high pathological complete remission (pCR) rate in neoadjuvant treatment (NAT). TNBC patients who achieve pCR have superior outcome than those without pCR. A meta-analysis was done to evaluate whether integrating novel approaches into NAT can improve the pCR rate in TNBC. Medical subject heading terms (Breast Neoplasm) and key words (triple negative OR estrogen receptor (ER) negative OR HER2 negative) AND (primary systemic OR neoadjuvant OR preoperative) were used to select eligible studies. Experimental arm in each study was considered as the testing regimen, and control arm was defined as the standard regimen in this meta-analysis. A total of 11 studies with 14 paired regimens were included in the final analysis. Aggregate pCR rate was 37.3% and 44.6% in the standard and testing group, respectively. Novel approaches in the testing regimen significantly improved the pCR rate in NAT of TNBC patients compared with the standard regimen, with an odds ratio (OR) of 1.34 (95% confidence interval (CI) 1.11–1.62, *P* = 0.002). Considering specific regimens, we demonstrated the pCR rate to be much higher in the carboplatin-containing (OR = 1.80, 95% CI 1.39–2.32, *P*<0.001) or bevacizumab-containing regimens (OR = 1.36, 95% CI 1.11–1.66, *P* = 0.003) than in the control regimens. The addition of carboplatin in NAT had a pCR rate as high as 51.2% in TNBC patients, with an absolute pCR difference of 13.8% as compared with control regimens. No significant heterogeneity was identified among studies evaluating the addition of carboplatin or bevacizumab efficacy in NAT. This meta-analysis indicates that these novel NAT regimens have achieved a significant pCR improvement in TNBC patients, especially among patients treated with carboplatin-containing or bevacizumab-containing regimen. This can help us design appropriate trials in the adjuvant setting and guide clinical practice.

## Introduction

Neoadjuvant treatment (NAT) is a well-established treatment approach for locally advanced breast cancer [1]. While, in early operable breast cancer, NAT shows equivocal efficacy compared with adjuvant therapy. A putative advantage of effective NAT could significantly increase the breast conserving surgery rate and also provide prognostic information. This would guide subsequent individual treatment [2]–[3], because patients achieving pathological complete remission (pCR) after NAT have more favorable outcomes than those without pCR [4].

Several different subtypes of breast cancer, including luminal A, luminal B, HER2+, basal like, and normal breast like subtype, have been identified by microarray data [5]–[6]. Immunohistochemical (IHC) status of estrogen receptor (ER), progesterone receptor (PgR), HER2, and Ki67, has been adapted to construct molecular breast cancer subtype which can be used to make therapeutic choices [7]. Regarding NAT and patients with triple negative breast cancer (TNBC), defined as ER-/PgR-/HER2-, such patients have a higher pCR rate than non-TNBC patients, while those TNBC patients who fail to achieve pCR after NAT have a poor prognosis [8]. The Collaborative Trials in Neoadjuvant Breast Cancer (CTNeoBC) working group has embarked upon a large meta-analysis, including more than 12,000 patients enrolled in published trials, to evaluate the relationship between pCR and patient’s outcome. Importantly, this study demonstrated that pCR is most likely to predict clinical benefit in TNBC and HER2 positive breast cancer patients [9]. Furthermore, Food and Drug Administration (FDA) has made an agreement to use pCR as an endpoint for accelerated new drug approval in high risk early breast cancer, leading to the approval of pertuzumab in NAT of HER2+ breast cancer [10].

At present, the standard NAT regimen for TNBC usually includes 4–8 cycles of taxanes and anthracyclines, producing a pCR rate of ∼30% [7]–[8]. Integrating anti-HER2 agents into NAT has nearly doubled the pCR rate compared with chemotherapy alone in HER2+ breast cancer patients [11]–[13]. To further improve pCR rate in HER2- patients, several other chemotherapeutic drugs, including carboplatin [14]–[16], gemcitabine [17], capecitabine [18]–[19], and ixabapilone [20], have been tested in clinical trials. In addition, taxane-first sequencing [17], [21] and response-guided therapy strategies [22]–[23] were also evaluated in NAT. Furthermore, several other novel clinical trials have been designed to find out whether the addition of biological agents, including bevacizumab [24]–[25], zoledronic acid [26], and everolimus [27], can improve the pCR rate in TNBC patients. However, there are relatively few randomized controlled trials (RCT) only enrolling TNBC patients to test the above approaches. A wide variability in pCR comparison existed among studies due to the heterogeneity in patient characteristics, patient number, pCR definition, and study type. Indeed, some of these could not demonstrate a pCR difference because of a relatively small TNBC patient sample size.

Considering the benefits from NAT and the unmet medical needs for TNBC patients, there is a growing body of evidence suggesting that a pCR is beneficial using carboplatin or bevacizumab. It is, therefore, necessary to conduct a meta-analysis to systematically evaluate the effect of these novel approaches compared with the standard regimen in NAT of TNBC patients. We also aim to find out which specific regimen can improve pCR rate in TNBC patients to guide our further clinical practice and adjuvant trial designs.

## Methods

### Literature search and criteria for eligible studies

The Pubmed database was used to identify abstracts of articles involving human subjects. Online abstracts from the following conferences were also included: proceedings of Annual Meetings of the American Society of Clinical Oncology (ASCO 2008–2013), European Society of Medical Oncology Conference (ESMO, 2008–2013) and San Antonio Breast Cancer Symposium (SABCS, 2008–2013). Medical subject heading (MeSH) terms (Breast Neoplasm) and the following keywords were used to search: (1) triple negative OR ER negative OR HER2 negative; (2) breast cancer; and (3) primary systemic OR neoadjuvant OR preoperative. Two authors (X.-S. Chen, Y. Yuan) carried out literature searches and identified eligible articles based on the above standards. Ineligible studies were excluded, and discrepancies were resolved by discussion and consensus. For relevant studies, full articles, abstracts, or conference presentation slides were examined. Reference lists from articles were searched. Social sciences citation index (2008 to present) of Web of Science was also used to search papers citing eligible studies.

The following inclusion criteria were adopted to minimize variables that might introduce bias or explain heterogeneity of results: (1) full articles published online between January 2001 and December 2013; (2) conference abstracts were presented between January 2008 and December 2013; (3) original studies written in English; (4) at least 30 TNBC breast cancer women enrolled in RCT; (5) at least four cycles of anthracycline and taxane containing regimens in the control group; (6) sufficient data to extract the number and (re)construct a 2×2 contingency table to be labeled with number of total patients and patients achieved pCR after NAT, which was defined as no invasive tumor in the breast and axillary; (7) largest or most recent articles within reports including overlapped patients.

### Data extraction and quality assessment

Two independent investigators (X.-S. Chen, Y. Yuan) reviewed the quality of each study and extracted data on year of publication, study type, NAT regimen, number of TNBC patients treated with NAT, number of TNBC patients achieving pCR, NAT cycles, TNM stage, and the pCR definition. Disagreement was resolved by discussion or by one senior investigator (K.-W. Shen).

### Data synthesis and statistical analysis

For each study, we constructed a 2×2 contingency table for pCR rate for paired regimens in each study. pCR rate was systematically estimated and compared between control and experimental groups. Higgins I-square (*I^2^*), an index for heterogeneity, was also calculated: *I^2^* = (*Q*−[*k*−1])/*Q*×100%, where *Q* is the χ^2^ value of heterogeneity and *k* is the number of studies included. Along with *P*<0.05 for heterogeneity, *I^2^*>50% further indicates heterogeneity between studies. Funnel plot was calculated to investigate potential publication bias. In order to explain possible heterogeneity across studies, we further performed subgroup analyses. Odds ratio (OR) for the outcome measures between treatment arms and the 95% confidence interval (CI) for each eligible study group were evaluated. Fixed effects (Mantel–Haenszel) or random effects (Der Simonian and Laird) models were used based on whether heterogeneity was present between studies. RevMan v 5.2 (Copenhagen: The Nordic Cochrane Centre, The Cochrane Collaboration) was applied for statistical analysis, and statistical significance was set at *p*<0.05.

## Results

### Description of enrolled studies

**Figure 1:**
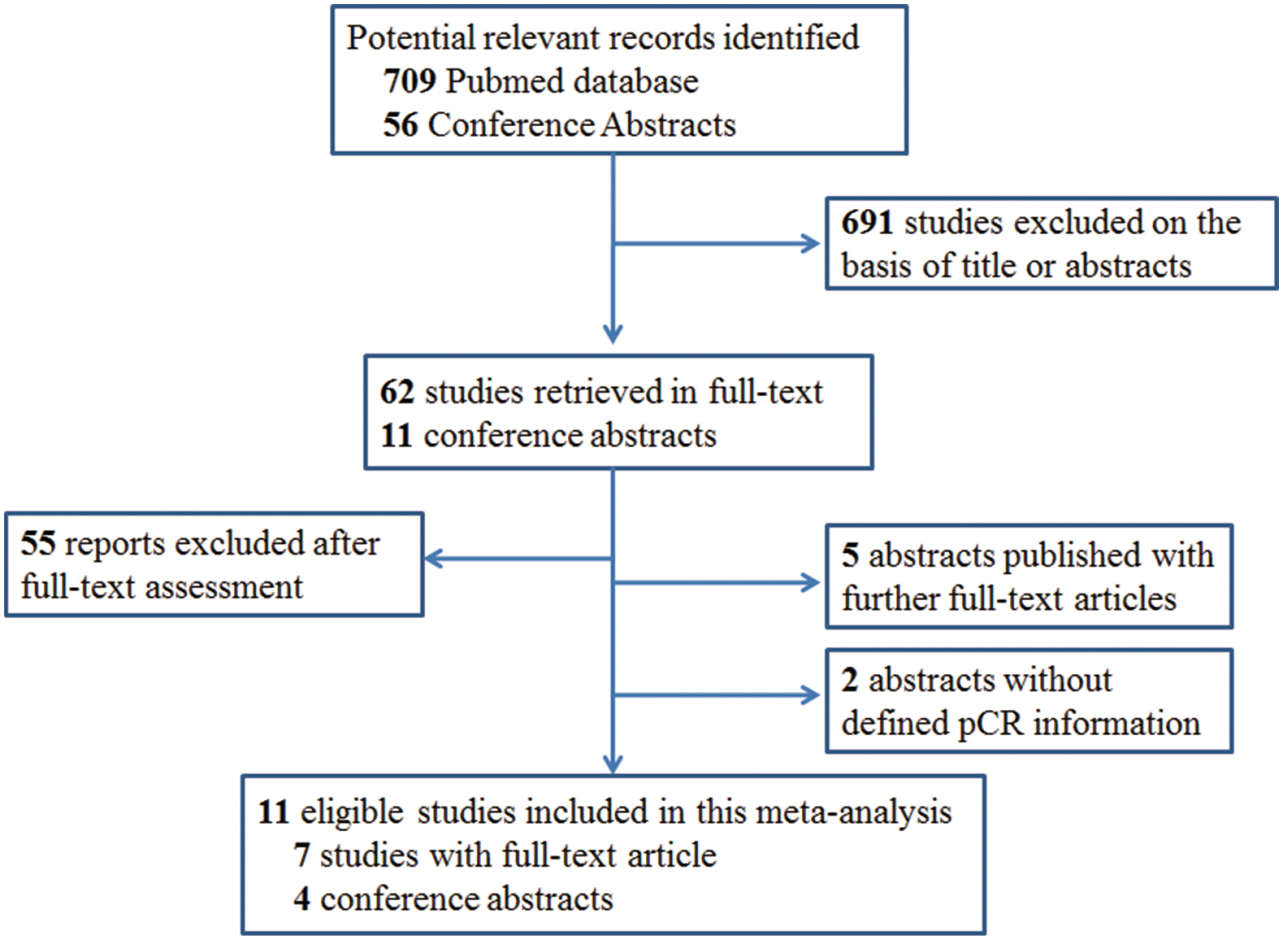
Study flow chart diagram for reports selection.

Seven hundred and nine potential full-text articles and 56 conference abstracts were identified. Regarding conference abstracts, 45 studies were single arm or historically controlled studies [28] or were unavailable to extract information to calculate the pCR rate in TNBC patients, while 5 studies were published subsequently with full-text articles and 2 studies didn’t provide the required pCR information for final analysis [29]–[30]. Ultimately, 11 independent studies were selected having a total of 2942 patients, 7 of which were from peer-reviewed reports and 4 from conference abstracts. Fig. 1 shows the study flow chart used to select eligible studies in the meta-analysis.

The Gepartrio study [23] had two parallel response and non-response arms according to ultrasound evaluation after two cycles of NAT; this was considered as two separate studies in our analysis. Two trials were designed as a 2×2 factorial study: the Neo-tAnGo trial [17] evaluated the effects of the addition of gemcitabine and paclitaxel-first in NAT; the CALGB 40603 study [31] was designed to demonstrate whether adding carboplatin or bevacizumab to standard NAT regimen would improve the pCR rate. Two treatment comparisons were extracted from each study and listed as a separate study in the final calculation. A total of 14 paired regimes were finally analyzed: carboplatin (5 studies), bevacizumab (3 studies), response-guided treatment (2 studies), agents sequencing (1 study), and 1 study each for gemcitabine, capcetabine and ixabepilone; 10 studies were from prospective RCT subgroup analyses, and 4 from RCT studies. Regarding agent sequencing, we used the original study definition with an anthracycline followed by taxane as the control regimen, while the reverse one as the experimental regimen. All control group regimens in each study were considered as the standard regimen, and the experimental group regimens were defined as the testing regimen in the final evaluation. Table 1 summarizes the main characteristics of all eligible studies.

**Table 1:** Baseline characteristics of included studies.

Study	Year	No. ofPts	TumorStage	Typeof Study	Regimen		pCR rate	
					Standard	Testing	Standard	Testing
**GeparTrio-R ** [23]	2010	284	T≥2 cm	Subgroupin RCT	TAC	TAC	50.3%	46.7%
**GeparTrio-NR ** [23]	2010	89	T≥2 cm	Subgroupin RCT	TAC	TAC → NX	12.5%	4.9%
**GEICAM/2006-03 ** [16]	2012	94	T>2 cm or N+	RCT	EC → T	EC → T + Cb	30.4%	29.8%
**NSABP B-40 ** [24]	2012	490	T≥2 cmN0-2a	Subgroupin RCT	T/TX/TG→AC	(T/TX/TG→AC) + Bev	39.8%	42.6%
**GeparQuinto ** [25]	2012	663	T>2 cm or N+	Subgroupin RCT	EC → T	(EC → T) + Bev	32.9%	43.3%
**ABCSG-24 ** [19]	2013	127	T1-4cN0-3	Subgroupin RCT	ET	ET + X	28.6%	40.6%
**Saura C, et al. ** [20]	2013	144	T2-3N0-3	Subgroupin RCT	AC → wP	AC → Ixa	40.8%	34.2%
**Neo-tAnGo-G ** [17]	2013	157	T2-4N0-3	Subgroupin RCT	EC & P	EC & P + G	31.5%	32.1%
**Neo-tAnGo-Seq ** [17]	2013	157	T2-4N0-3	Subgroupin RCT	EC → P/PG	P/PG → EC	28.8%	35.1%
**GeparSixto ** [32]	2013	315	T2-4 or T1c-4N+	Subgroupin RCT	PMBev	PMBev + Cb	42.7%	57.0%
**I-SPY 2 ** [33]	2013	56	T≥2.5 cm	Subgroupin RCT	wP → AC	wP + Vel/Cb → AC	26.1%	51.5%
**CALGB 40603-Cb ** [31]	2013	433	II–III, no T4d	RCT	(wP → AC) +/−Bev	(wP + Cb → AC) +/−Bev	41.0%	54.3%
**CALGB 40603-Bev ** [31]	2013	433	II–III, no T4d	RCT	wP+/−Cb → AC	(wP+/−Cb → AC) + Bev	44.0%	52.1%
**Zhang P, et al. ** [34]	2013	91	II–III	RCT	PE	PCb	14.0%	38.6%

Abbreviations: A, adriamycin; Bev, bevacizumab; C, cyclophosphomide; Cb, carboplatin; E, epirubicin; F, fluorouracil; G, gemcitabine; Ixa, Ixabepilone; M, non-pegylated-liposomal doxorubicin; N, navelbine; NA, non-available; No., number; NR, non-response; P, paclitaxel; pCR, pathological complete remission; Pts, patients; R, response; RCT, randomized controlled Trial; Seq, sequence; T, docetaxel; Vel, veliparib; wP, weekly paclitaxel; X, capacitabine.

### Comparison pCR rate among regimens

**Figure 2:**
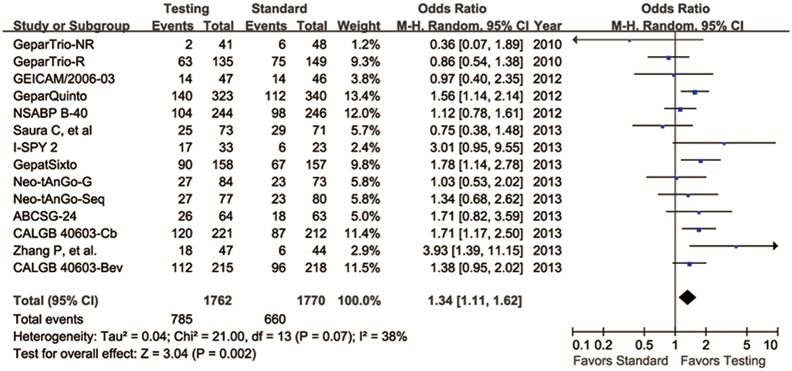
Forest plot of frequency of pCR in all eligible studies. Squares indicate point estimate of each study. Size of square indicates relative contribution of each study. Solid horizontal lines represent 95% CIs. Diamond indicates pooled OR value.

With the pCR definition as no invasive tumor in the breast and axillary, pCR rate ranged from 12.5% to 50.3% in the standard arm, and was between 4.9% and 57.0% in the testing arm. The aggregate pCR rate was 37.3% (660/1770) and 44.6% (785/1762) in the standard and testing group, respectively. Compared with the standard treatment arm, the probability of achieving a pCR was significantly higher in the testing arm (OR = 1.34, 95% CI 1.11–1.62, *p* = 0.002) (Fig. 2).

**Figure 3:**
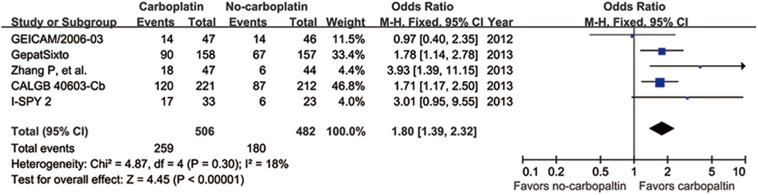
Forest plot of frequency of pCR: carboplatin versus no-carboplatin. Squares indicate point estimate of each study. Size of square indicates relative contribution of each study. Solid horizontal lines represent 95% CIs. Diamond indicates pooled OR value.

Five studies enrolled 988 TNBC patients to evaluate whether the addition of carboplatin in NAT would improve the pCR rate. The absolute pCR rate was 37.3% (180/482 patients) in the no-carboplatin-containing arm compared with 51.2% (259/506 patients) in the carboplatin-containing arm. The probability of achieving a pCR was much higher for the carboplatin-containing arm for the no-carboplatin group (OR = 1.80, 95% CI 1.39–2.32, *p*<0.001) (Fig. 3).

**Figure 4:**
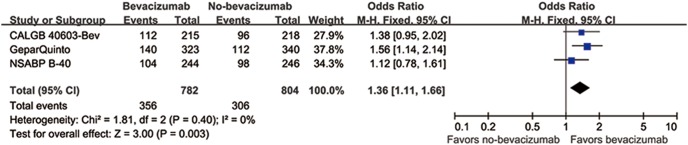
Forest plot of frequency of pCR: bevacizumab versus no-bevacizumab. Squares indicate point estimate of each study. Size of square indicates relative contribution of each study. Solid horizontal lines represent 95% CIs. Diamond indicates pooled OR value.

There were three studies (1586 patients) available to compare the efficacy with or without bevacizumab. The absolute pCR rate was 38.1% (306/804) and 45.5% (356/782) in the control and bevacizumab-containing arm, respectively. There was a statistically significant difference in terms of pCR rate with a higher pCR rate in bevacizumab-containing regimens (OR = 1.36, 95% CI 1.11–1.68, *p* = 0.003) (Fig. 4).

Two parallel studies in the Gepartrio trial demonstrated that response-guided NAT regimen selection in TNBC patients didn’t improve the pCR rate compared with the standard one (OR = 0.80, 95% CI 0.51–1.25, *p* = 0.33) (Fig. S1). Regarding agent sequencing of NAT regimen, no statistically significant pCR improvement was observed with taxane followed by anthracycline regimen compared with the standard (OR = 1.34, 95% CI 0.68–2.62, *p* = 0.40). Moreover, adding either gemcitabine or capecitabine to standard NAT regimen did not improve the pCR rate (*P*>0.05). Compared with paclitaxel, ixabapilone failed to increase pCR rate in TNBC patients (*P*>0.05).

### Heterogeneity and subgroup analysis

**Figure 5:**
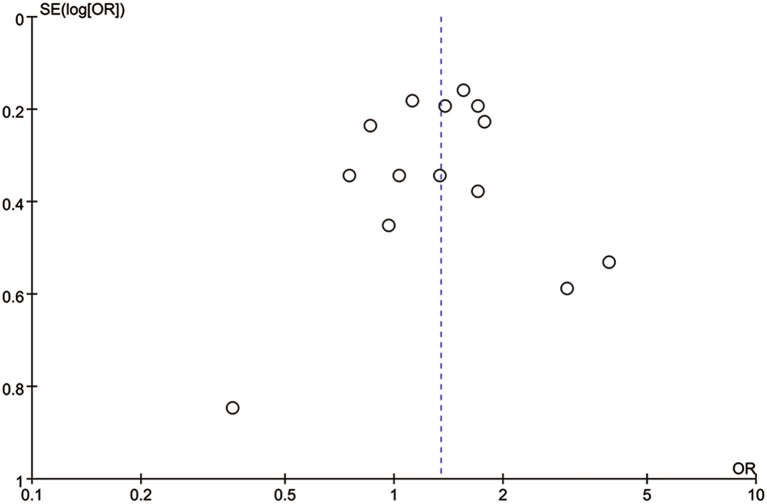
Funnel plot analysis of potential publication bias for all eligible studies. Abbreviate: OR, Odds Ratio; SE, Standard Error.

*I* ^2^ was used to calculate the between-study heterogeneity. The value was 38% in whole population analysis, while the *P* value was 0.07, indicating there was no obvious heterogeneity among these studies. Similarly, no heterogeneity was identified for studies evaluating the efficacy of carboplatin or bevacizumab in TNBC patients (*P>*0.05 and *I* ^2^<50%). Moreover, subgroup analysis was applied to evaluate the impact of different treatment regimen (carboplatin vs. bevacizumab vs. no-carboplatin or bevacizumab), study type (subgroup in RCT vs. RCT), or publication type (full-text article vs. abstract) to the pooled results. Statistical significance existed among different treatment regimen subgroups (*P* = 0.009) and among publication type subgroups (*P* = 0.01). Regarding treatment regimen, carboplatin subgroup had a relative higher OR value than subgroup with no-carboplatin or bevacizumab regimen (*P* = 0.002). There was no significant difference in OR value between carboplatin subgroup and bevacizumab subgroup (*P* = 0.09, Fig. S2). For studies only published with abstract, the OR value was 1.73 (95% CI 1.35–2.21), which was much higher than studies with full-text article published (OR = 1.15, 95% CI 0.93–1.41) (Fig. S3). Funnel plot analysis showed there was no publication bias in the whole population (Fig. 5). However, a publication bias was found in carboplatin subgroup and full-text article subgroup, where studies missed from the bottom left quadrant (Figs. S4–S5). It could be speculated that some unpublished studies with an OR of approximately 1 but were not published because of their negative results.

## Discussion

Adjuvant systemic therapy has significantly decreased breast cancer recurrence and mortality rate [35]–[37]. Sequencing of chemotherapy and surgery did not alter distant relapse free survival outcome, favoring NAT administration. This has been increasingly considered as an excellent platform to test novel therapeutic regimens, especially in TNBC or HER2+ diseases [10]. For TNBC patients, due to lack of available targeting agents, chemotherapy is the only choice in NAT. This routinely contains anthracycline and taxane agents, with pCR rate ∼30% [7]–[8]. Moreover, pCR rate improvement in TNBC patients may be associated with disease recurrence rate reduction [8], [38]. Therefore, it is crucial to find out which novel approach can significantly increase the pCR rate in TNBC patients, further guiding NAT and adjuvant trial design.

This meta-analysis summarizes the available evidence on the testing of new approaches in NAT of TNBC patients and demonstrates the superiority of the testing regimens which integrate novel approaches, in terms of pCR improvement, compared with standard regimens (OR = 1.34). A wide variability in the range of pCR rate was observed among the studies included in our analysis, mainly due to the Gepartrio study, which included patients who failed to respond after two cycles of NAT resulting in pCR rates of only 12.5% and 4.9% in the control and testing arms. Regarding the use of specific agents, our data supports carboplatin-containing (OR = 1.80) or bevacizumab-containing (OR = 1.36) regimens that achieved statistically higher pCR rates, as was the case with the phase II CALGB 40603 study. Moreover, our meta-analysis included a larger number of TNBC patients allowing to draw more robust conclusions compared to individual phase II studies. Furthermore, we demonstrated that pCR improvement with additional carboplatin or bevacizumab treatment was not associated with a specific controlled chemotherapy regimen, which may reflect its common biological behavior and de novo sensitivity of TNBC to carboplatin or bevacizumab treatment.

TNBC is characterized biologically by having a histopathological similarity with germline BRCA1-mutated breast cancer, with 90% of BRCA1-mutation tumors being considered as TNBC [39]. Due to its deficiency of DNA repair mechanisms, BRCA1 mutation-associated TNBC cells are particularly sensitive to DNA-damaging platinum agents, like cisplatin or carboplatin [40]. A phase II study evaluated cisplatin monotherapy as NAT in TNBC patients, showing a pCR rate of 22% [41]. For breast cancer patients with BRCA1 mutation, single-agent cisplatin NAT can achieve an extremely high pCR rate of 83% [42]. Several phase II single-arm studies have tested the combination of taxanes and platinum salts as NAT for TNBC patients, with pCR rates of 33–77%, indicating that platinum salts are especially active in TNBC treatment [40]. Our findings are consistent with previous phase II studies showing that adding carboplatin to NAT regimens can achieve a pCR rate up to 51.2%. Compared with the no-carboplatin-containing standard NAT regimen, the probability of achieving a pCR was much higher in the carboplatin-containing arm (OR = 1.80). The CTNeoBC study, as well as the Germany Breast Group’s meta-analysis, showed that pCR was a strong surrogate endpoint for survival in TNBC tumors [9], [38]. Recently, Hatzis C, et al., reported that there was a linear dependence of the 5-year disease free survival (DFS) on the pCR rate by bootstrap analysis in NAT of TNBC patients, with a slope of 0.47 indicating a 4.7% improvement in DFS for every 10% increase in pCR rate [43]. In our current meta-analysis, carboplatin-containing regimen was 13.8% higher in terms of pCR rate than the standard group. This would theoretically show a 6.5% increase in 5-year DFS in TNBC patients.

Activated angiogenesis-related genes and high vascular endothelial growth factor (VEGF) expression are frequently present in TNBC tumors, indicating anti-angiogenesis may be a potential effective strategy in NAT [44]. Bevacizumab, a VEGF-A inhibitor, has shown great efficacy in combination with chemotherapy in patients with HER2- metastatic breast cancer, especially in TNBC patients [45]–[46]. In the neoadjuvant setting, the NSABP B-40 and GeparQuinto trials have demonstrated that bevacizumab could increase the pCR rate in NAT of HER2- patients [25]. However, subgroup analysis showed that the pCR improvement in the GeparQuinto trial was restricted to TNBC patients [25], while the greatest bevacizumab effect was only seen in the non-TNBC patients in the NSABP B-40 trial [24]. In the current meta-analysis, we included we included subgroup data from the above two trials and from the CALGB 40603 study and were able to show a significant pCR difference favoring the addition of bevacizumab in NAT of TNBC (OR = 1.36). However, due to severe life-threatening toxicities [47] and outcome improvement failure with bevacizumab in an adjuvant trial [48], in our opinion, we must still wait for more robust results before integrating bevacizumab in routine clinical NAT of TNBC patients.

Preclinical data has demonstrated there is a cross-resistance effect dependent on the sequence of anthracyclines and taxanes treatment in breast cancer [49], suggesting that early administration of taxanes may improve patient outcome. A retrospective study showed that taxane-first sequencing of (neo) adjuvant treatment was associated with lower risk of relapse and death [21]. A prospective RCT was designed to determine whether taxane-first sequencing regimen for NAT was able to improve pCR rate in breast cancer patients [17]. However, for TNBC patients included in our meta-analysis, the taxane-first regimen did not significantly improve the pCR rate, although this may be due to the efficacy difference within molecular subtypes and the relatively small number of included patients [50].

NAT is an ideal platform to test the response and chemo-sensitivity in vivo, thus guiding further treatment. Two parallel studies from the GeparTiro trial showed that the response-guided regimens could not obtain a higher pCR rate than the standard [51]–[52]. Furthermore, follow-up data demonstrated there was a survival advantage in Luminal A and Luminal B subtypes but not in triple negative or HER2+ subtypes among patients treated with response-guided regimens [38] The main reason may be due to the low pCR rate difference in TNBC patients, which was also confirmed in our study.

Several limitations in our meta-analysis should be discussed. Firstly, a majority of included studies were from subgroup data in RCTs, leading to insufficient details of patient characteristics and toxicity for TNBC patients. Our meta-analysis was not able to determine which TNBC subgroup of TNBC could derive more benefit from the testing regimen and whether new agents would increase the chance of severe side events. Additionally, we included several studies which were only published as abstracts. This could be associated with the Tower of Babel bias, meaning that positive results would be highly presented in international conferences and negative studies left out. Furthermore, the 2×2 factorial study in our meta-analysis was considered as two independent paired regimens, perhaps leading to the cross-talk treatment efficacy interaction [17], [31]. Moreover, the I-SPY2 trial also concurrently used veliparib with carboplatin in the testing group, which could overestimate carboplatin efficacy [33]. Currently, the control NAT regimens in our selected studies are no longer viewed as standard due to the inferior efficacy in NAT or adjuvant treatment setting, such as the every 3 weeks paclitaxel [17], [34] and 4 cycles of epirubicin plus docetaxel regimen [19].

## Conclusion

Our meta-analysis showed a significant pCR rate improvement in TNBC patients treated with tnovel carboplatin or bevacizumab-containing regimens. In addition, the pCR rate was an absolute 13.8% higher in the carboplatin-containing regimen compared with the standard, indicating that these carboplatin-containing NAT regimens deserve further evaluation also in the adjuvant setting to determine if this pCR improvement will translate into a survival benefit.

## Supporting Information

Figure S1 Forest plot of frequency of pCR: response-guided. Squares indicate point estimate of each study. Size of square indicates relative contribution of each study. Solid horizontal lines represent 95% CIs. Diamond indicates pooled OR value.
(TIF)

Figure S2 Subgroup analysis according to treatment regimen: carboplatin vs. bevacizumab. Squares indicate point estimate of each study. Size of square indicates relative contribution of each study. Solid horizontal lines represent 95% CIs. Diamond indicates pooled OR value.
(TIF)

Figure S3 Subgroup analysis according to publication type: full-text article vs. abstract. Squares indicate point estimate of each study. Size of square indicates relative contribution of each study. Solid horizontal lines represent 95% CIs. Diamond indicates pooled OR value.
(TIF)

Figure S4 Funnel plot analysis of potential publication bias for subgroups with different treatment regimen. Abbreviate: OR, Odds Ratio; SE, Standard Error.
(TIF)

Figure S5 Funnel plot analysis of potential publication bias for subgroups analysis: full-text article vs. abstract. Abbreviate: OR, Odds Ratio; SE, Standard Error.
(TIF)

Checklist S1 PRISMA Checklist.
(DOC)
