# Supplementary figures and images for: Both Carboplatin and Bevacizumab Improve Pathological Complete Remission Rate in Neoadjuvant Treatment of Triple Negative Breast Cancer: A Meta-Analysis
Source: PLoS One. 2014 Sep 23;9(9):e108405. doi: 10.1371/journal.pone.0108405 (PMC4172579; doi:10.1371/journal.pone.0108405)

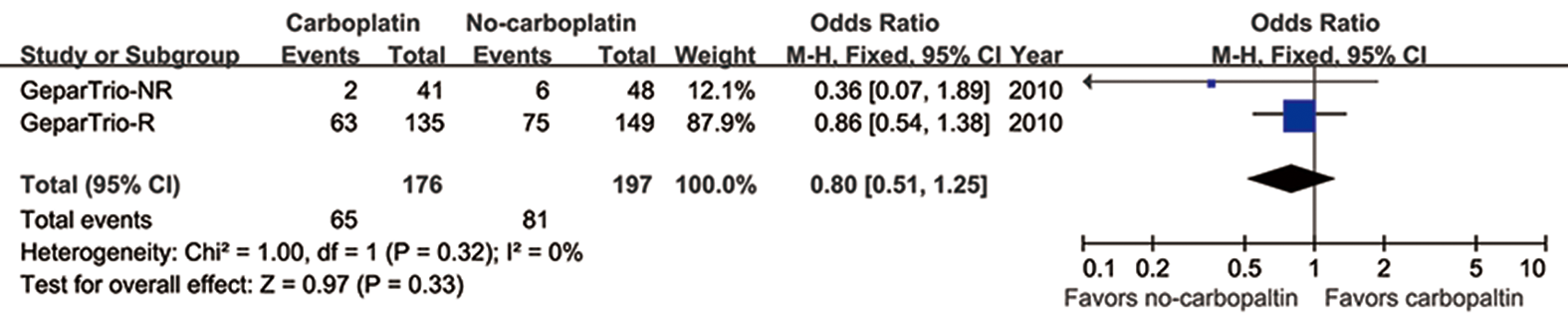

Supplement: Figure S1 — Forest plot of frequency of pCR: response-guided. Squares indicate point estimate of each study. Size of square indicates relative contribution of each study. Solid horizontal lines represent 95% CIs. Diamond indicates pooled OR value. (TIF) [file pone.0108405.s001.tif]

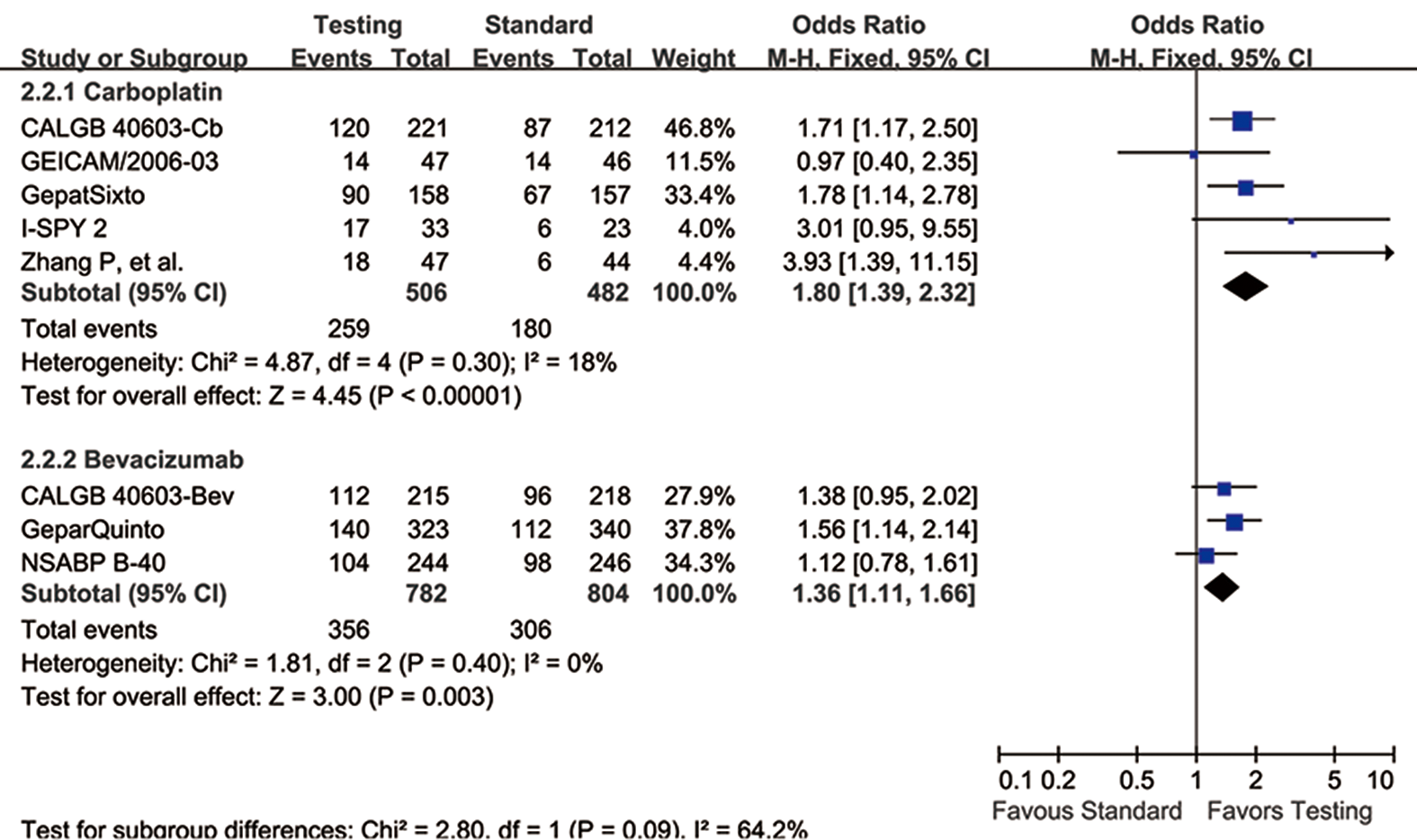

Supplement: Figure S2 — Subgroup analysis according to treatment regimen: carboplatin vs. bevacizumab. Squares indicate point estimate of each study. Size of square indicates relative contribution of each study. Solid horizontal lines represent 95% CIs. Diamond indicates pooled OR value. (TIF) [file pone.0108405.s002.tif]

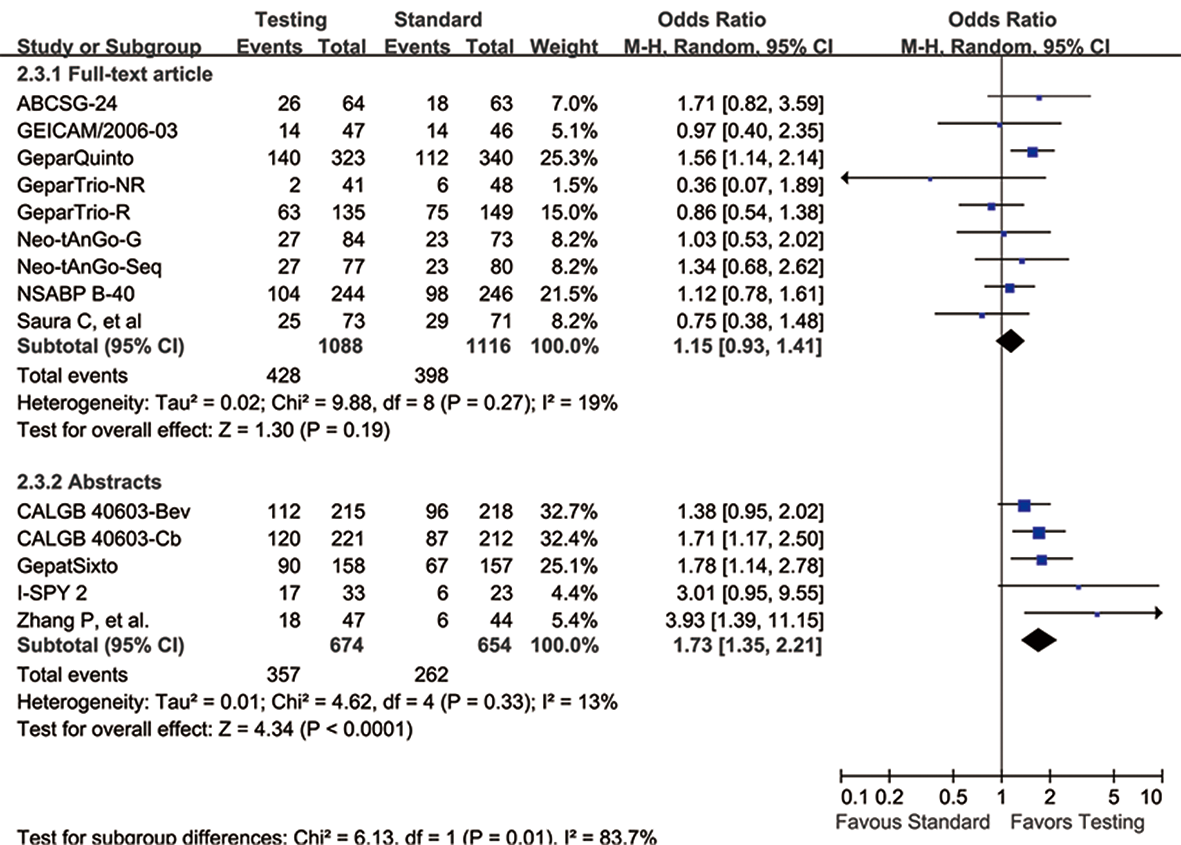

Supplement: Figure S3 — Subgroup analysis according to publication type: full-text article vs. abstract. Squares indicate point estimate of each study. Size of square indicates relative contribution of each study. Solid horizontal lines represent 95% CIs. Diamond indicates pooled OR value. (TIF) [file pone.0108405.s003.tif]

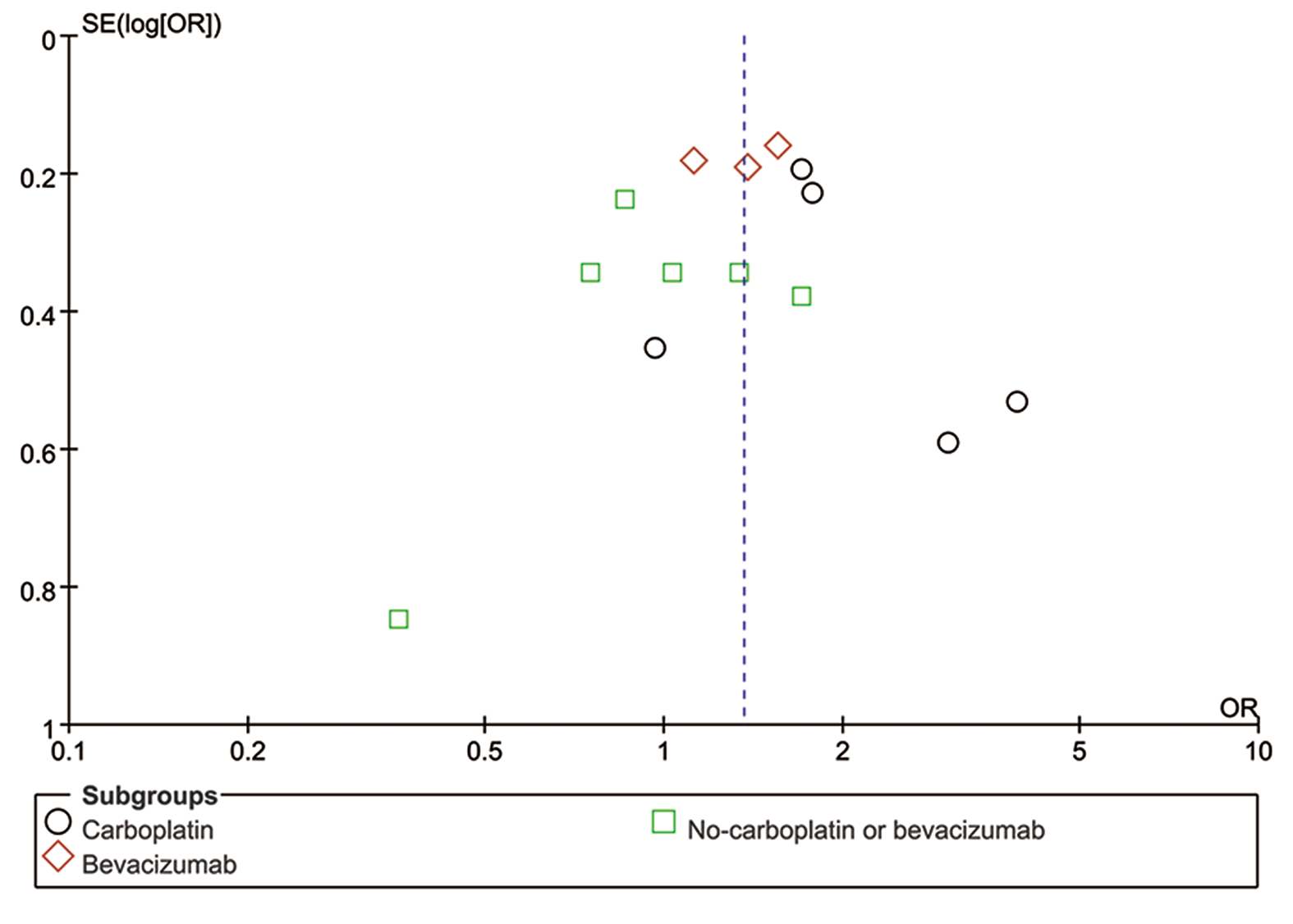

Supplement: Figure S4 — Funnel plot analysis of potential publication bias for subgroups with different treatment regimen. Abbreviate: OR, Odds Ratio; SE, Standard Error. (TIF) [file pone.0108405.s004.tif]

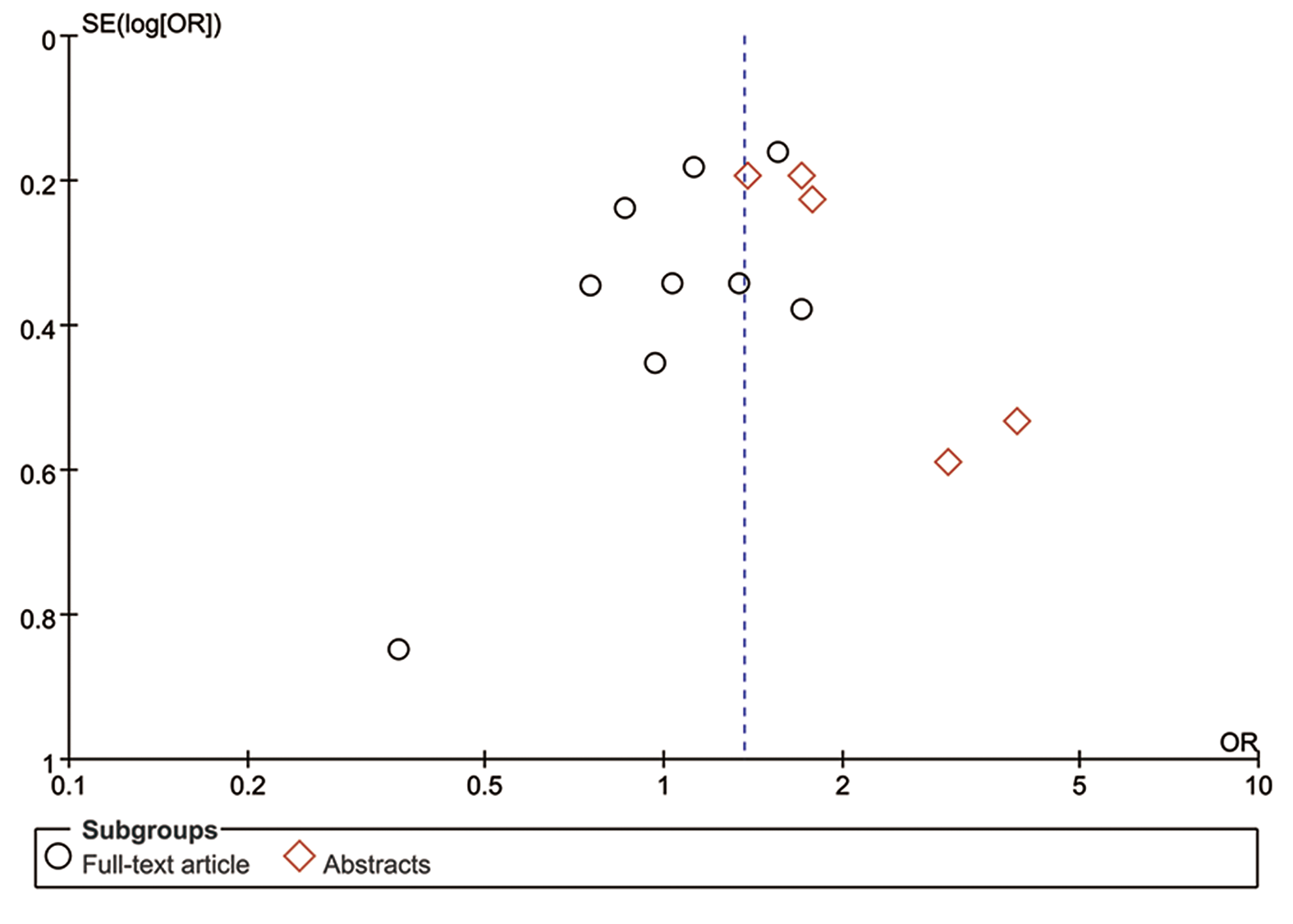

Supplement: Figure S5 — Funnel plot analysis of potential publication bias for subgroups analysis: full-text article vs. abstract. Abbreviate: OR, Odds Ratio; SE, Standard Error. (TIF) [file pone.0108405.s005.tif]
